# Research on Linear Energy Transfer of SiC Materials Based on Monte Carlo Method

**DOI:** 10.3390/mi16101092

**Published:** 2025-09-26

**Authors:** Jiamu Xiao, Heng Xie, Shougang Du, Shulong Wang, Tianlong Zhao, Hongxia Liu

**Affiliations:** 1 Wide Bandgap Semiconductor Technology Disciplines State Key Laboratory, Xidian University, Xi’an 710071, China; mu_xiaojm@aliyun.com (J.X.); sgdu@stu.xidian.edu.cn (S.D.); zhaotl@xidian.edu.cn (T.Z.); hxliu@mail.xidian.edu.cn (H.L.); 2 Nanjing Electronic Equipment Institute (NEEI), Nanjing 211103, China; muyi2012xh@163.com; 3 National Key Laboratory of Air-Based Information Perception and Fusione, Luoyang 471000, China

**Keywords:** linear energy transfer, SiC, monte carlo, SET

## Abstract

The energy deposition process for the main components of SIC Schottky diodes is simulated based on Geant4. Particle bombardment results were simulated under different angles, target materials and doping concentrations on the same target material for different light particles and heavy ions, and then the Linear Energy Transfer of SiC materials and external conditions that affect LET are obtained. The results show that the LET value of protons exhibits significant oscillations at low energy incidence, gradually decreasing exponentially after 10^−1^ MeV. Alpha particles have a LET peak near 1 MeV, while beta particles show an exponential decrease. The LET values at low energy levels increase exponentially, while at high energy levels, the LET values show a similar linear relationship with energy. For different incident angles, the average LET value of protons in the low-level region gradually increases as the incident angle increases. The average LET value of protons in the remaining energy ranges is less affected by angle; the incident angle has no significant effect on the LET distribution of alpha particles within the full spectrum range. The results provide important references for understanding the energy deposition process and LET distribution of silicon carbide devices under single-particle interaction.

## 1. Introduction

With the continuous progress of space technology, more and more electronic devices need to be applied in various radiation environments. As an important part of power electronic technology, the stability of power devices in a space radiation environment is particularly important. Therefore, a new research direction combining semiconductor materials, devices, and radiation physics has gradually gained importance with scientific researchers [1,2,3,4].

With the continuous advancement of space technology, an increasing number of electronic devices need to be applied in various radiation environments. As a crucial component of power electronics technology, the stability of power devices in space radiation environments is particularly important [5,6,7]. In 1993, the homoepitaxial growth of 4H-SiC materials using chemical vapor deposition (CVD) was first reported, with a mobility of more than 700 cm^2^∙V^−1^∙s^−1^. At the same time, Larkin et al. proposed the concept of “location-competition”, which greatly improved the control ability of SiC doping [8]. In 2008, researchers of Keri Company used junction terminal technology to manufacture JBS diodes with a breakdown voltage of up to 10 kV on wafers of more than 1.5 cm^2^ [9]. The excellent reverse breakdown characteristic of the JBS diode is fully demonstrated. In 2019, WuJiupeng et al. increased the surge resistance of JBS devices by 2~4 times by adding large area P-zone and P-type ohmic contact [10]. The remarkable progress in the growth and technology of SiC materials provides a good foundation for the high power application of SIC materials.

In 2015, Sabuhi Ganiyev et al. simulated the I–V characteristics of a Ni/4H-SiC Schottky diode induced by electron radiation with the energy of 3 MeV and a high temperature through a Silvaco computer-aided simulation tool based on thermal electron emission theory [11]. In 2017, A.A. Lebedev et al. studied the electrical characteristics of 4H-SiC Schottky diodes under electron irradiation with 0.9 MeV of energy [12]. Omotoso et al. carried out high-energy electron irradiation experiments on low-doped N-type 4H-SiC Schottky diodes with Ni-based Schottky metal [13]. In 2019, P.S. Gromova et al. experimentally studied the probability of the single event burnout of several 4H-SiC Schottky power diodes induced by heavy ions under different bias voltages [14]. The impact of bias voltage on the radiation effect of silicon carbide metal-oxide-semiconductor field-effect transistors (SiC MOSFETs) was studied using 1-MeV high-energy electrons [15]. Pavel Hazdra et al. used deep-level transient spectroscopy (DLTS), current–voltage (I-V) curves, and open-circuit voltage decay (OCVD) measurement methods to explain the different effects of displacement damage caused by neutron irradiation on the static characteristics of 4.5 kV silicon (Si) and 4H-silicon carbide (4H-SiC) P-i-N power diodes [16]. Rafi et al. fabricated PN-junction segmented diodes with a four-quadrant distribution on SiC epitaxial layers and high-resistivity bulk silicon substrates of 10 μm thickness. By analyzing the current–voltage (I-V) curves and capacitance–voltage (C-V) curves of the devices, the results show that under the experimental conditions of specific particle types and irradiation doses, the SiC devices exhibit a reverse current that is four orders of magnitude lower than that of the Si devices after irradiation [17].

Irradiation experiments on silicon carbide (SiC), especially in space radiation and nuclear radiation scenarios, often rely on large-scale radiation sources (such as particle accelerators, nuclear reactors, and isotope sources). Such equipment is associated with high operational costs (with a single experiment costing tens of thousands to hundreds of thousands of RMB) and long reservation cycles (some large-scale facilities require applications several months or even years in advance) [18,19]. Additionally, radiation poses safety risks to operators during experiments, and the destructive nature of radiation on samples means that only limited data can be obtained from a single experiment (for example, samples require offline testing after irradiation, making real-time tracking of dynamic processes impossible).

Geant4 can reproduce the entire process of “particle–material interaction” through digital simulation, without relying on physical radiation sources or sample consumption: by simply inputting the microparameters of silicon carbide (such as the atomic density, bandgap, and crystal structure) and radiation source parameters (such as particle type, energy, and flux), it can simulate physical processes under different irradiation conditions. This significantly reduces the economic and time costs of experiments while completely avoiding radiation safety risks. Combining the “energy deposition distribution inside the device” output by Geant4 with the “silicon carbide defect evolution model,” the performance degradation trends of the device under different radiation doses (e.g., the variation curve of reverse leakage current with dose, and the threshold voltage drift) can be predicted. This provides data support for the device’s “radiation-hardened design indicators” (e.g., the leakage current does not exceed 1 μA under a dose of 100 kGy) [20,21].

The energy deposition of charged particles is a crucial factor inducing single-event effects (SEEs). Energy deposition is a function of the product of particle displacement distance and Linear Energy Transfer (LET). This paper primarily models the main constituent materials of silicon carbide (SiC) Schottky diodes and conducts simulation experiments for energy deposition statistics using Geant4 software.

Experiments were carried out on the bombardment results of four types of light particles and ten types of heavy ions under multiple conditions: different incident angles, different target materials, and the same target material with different doping concentrations. The LET distribution curves across the full energy spectrum were extracted, and the external factors influencing LET were analyzed.

## 2. Device Model and Simulation

### Devices Under Test

**Figure 1:**
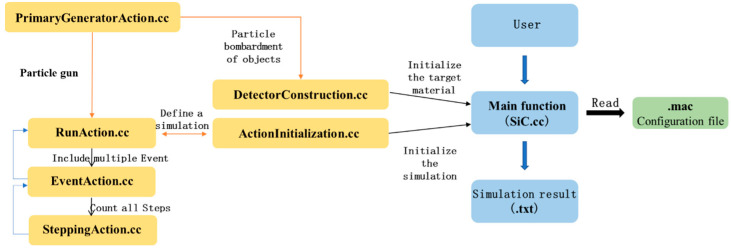
Simulation Program Call Flowchart.

The model of Geant4 (10.04) first requires the G4Box*solidWorld function to define the World, which is used as the space environment of the target material and fills the world with vacuum environment. The size of the device is 200 mm × 200 mm × 200 mm, which is a rectangular structure. The functions G4LogicalVolume*logicWorld and G4VPhysicalVolume*physWorld are used to assign the space environment to the logical volume and the physical process volume. The types of target materials used in the experiment are silicon carbide, silicon dioxide, titanium metal, and aluminum. Here, G4NistManager is used to select the components of the corresponding materials and the definition of material density ρ; Silica, titanium, and aluminum can be used in the material library defined in Geant4; SiC needs to be created by the proportion and density of material elements. The compiled .cc program files and their simulation calling sequence are shown in Figure 1.

The sensitive volume of the SiC device adopts the rectangular parallelepiped (RPP) structure and is used to characterize the sensitivity of the SiC device to the single-event effect. It has the following assumptions:

(1) The Linear Energy Transfer value in the sensitive volume is constant and does not change with the coordinate position.
(2) All particles shot into the sensitive body by the particle gun are completely absorbed by the sensitive volume, and the energy of the incident particles completely leads to the ionization process, resulting in electron–hole pairs.
(3) The electron–hole pairs generated in the sensitive space meet the threshold set by the physical model, which can be considered as a single event upset in the circuit.

**Figure 2:**
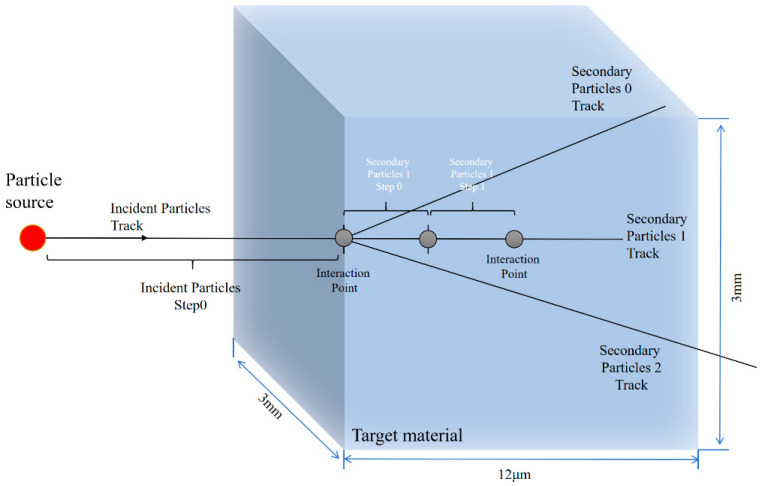
The structural model and dimensions of the SiC material in Geant4.

The structural model and dimensions of the SiC material in Geant4 are shown in Figure 2 and Table 1 as follows.

Since the ionization process between the particle and the target material is mainly simulated in this experiment, the FTFP_BERT_HP physical process is selected, and the default threshold cutoff is 0.7 mm. The particle gun used to emit particles is defined by the G4ParticleGun() function, and the number of particles emitted each time is set to 1. According to the composition of the cosmic radiation environment, particle sources are mainly divided into two categories: light particles and heavy ions. Light particles include protons, α particles, gamma particles, β particles, and neutrons. Heavy ions refer to ions heavier than α particles, and most of them are all element ions with atomic numbers ranging from 2 to 92. In this paper, 10 kinds of heavy ions including B11, C12, N14, O16, Ne20, Ar40, Fe56, Ni58, Kr84, and Bi209 are selected according to the atomic number order. The simulation carries out full energy spectrum scanning statistics for incident particles, and the scanning range is from 10^−3^ MeV to 10^7^ MeV. In order to prevent the overflow of computer memory caused by excessive data, the initial energy of particles is freely defined by macro text and the simulation is carried out step by step in different stages.

## 3. Results and Discussion

In this paper, four kinds of light particles and ten kinds of heavy ions with different atomic numbers are selected, respectively, to bombard the target materials. Since the space radiation environment covers almost all particle types and particle energies, this paper uses the full spectrum energy scanning from 10^−4^ MeV to 10^7^ MeV for different kinds of particles. In order to prevent memory overflow, the segmented scanning method of the dynamic step interval is adopted, and because the LET distribution result of single-particle incidence will have a large deviation due to the influence of probability distribution, 1000 particle experiments are carried out at the same energy. Based on 1000 repeated experiments, the LET distribution result is obtained and the arithmetic mean value is calculated to obtain the average LET value at this energy. The LET distribution of light particles and heavy ions on SIC material with a thickness of 12 μm is described below, and the particle incidence range is within 25% of the material center. The incidence angle is vertical.

A. Results on four light particles

**Figure 3:**
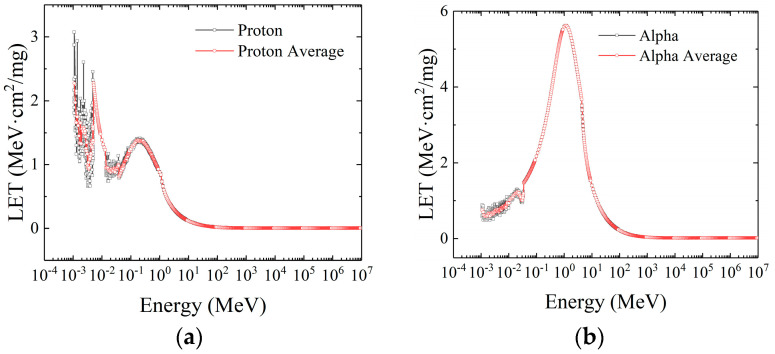
The LET distribution of proton (**a**) and alpha particles (**b**).

The LET distribution curve of protons is shown in Figure 3a. The abscissa in the figure is a logarithmic distribution with a base of 10. It can be seen that there is no numerical record of LET in the range of 10^−4^ MeV to 10^−3^ MeV, because this energy range is lower than the minimum simulation threshold of Geant4. Therefore, only particles with energy greater than 10^−3^ MeV can collide with the target material to generate an effective step, and then we calculate the energy statistics.

The LET distribution curve of protons is shown in Figure 3b. In the energy interval from 10^−3^ MeV to 10^−2^ MeV, the LET value of a proton has two peaks at both ends of the interval, with the average LET value being 2.272 MeV·cm^2^/mg and 2.277 MeV·cm^2^/mg, respectively. The average LET distribution within the interval first decreased to about 0.931 MeV·cm^2^/mg, and then increased rapidly. From 10^−2^ MeV, the average LET of protons first decreases to 0.813 MeV·cm^2^/mg, then increases to 1.374 MeV·cm^2^/mg, and then decreases exponentially. When the energy is about 510 MeV, the LET tends to be stable, at about 0.005 MeV·cm^2^/mg.

When low-energy particles are incident, the main reason for the high LET value is that the collision type between the proton and the target material is elastic collision, and the resulting secondary or multiple collisions are caused by the initial elastic scattering. Due to scattering, the movement distance of particles in the target material is larger than the thickness of the target material, which makes the energy deposition produce more electron–hole pairs, resulting in the high LET value.

The LET distribution of alpha particles is more regular than that of protons, and there are no frequent fluctuations. In the energy range of 10^−3^ MeV to 100 MeV, the average LET value gradually increases exponentially to the maximum of 5.620 MeV, with a low LET value of 1.010 MeV·cm^2^/mg within the range of 0.03 MeV. From 100 MeV, the LET value of alpha particles drops rapidly until 6800 MeV, and the LET value tends to be 0.016 MeV∙cm^2^/mg. The reason for this phenomenon is mainly because of the elastic scattering between particles.

**Figure 4:**
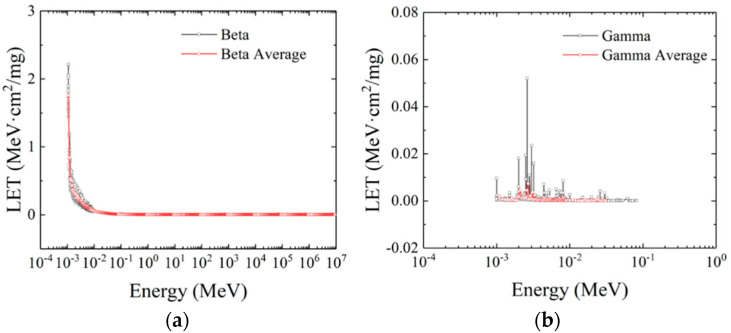
The LET distribution of beta (**a**) and gamma (**b**) particles.

The LET distribution curves of beta particles and gamma particles are shown in Figure 4a and Figure 4b, respectively. The LET value of beta particles is 1.759 MeV·cm^2^/mg at 10^−3^ MeV. With the gradual increase in the initial energy of the particles, the LET value decreases rapidly, and gradually stabilizes to 0.005 MeV·cm^2^/mg at 0. 39 MeV. The LET value of the gamma particle ranges from 10^−3^ MeV to 10^−1^ MeV, and there are two tiny peaks at 0.0021 MeV and 0.0026 MeV, respectively, 0.0046 MeV·cm^2^/mg and 0.0045 MeV·cm^2^/mg. The LET value in other energy ranges is 0, from which it can be considered that gamma particles directly penetrate the device.

B. Results on Heavy Ions

**Figure 5:**
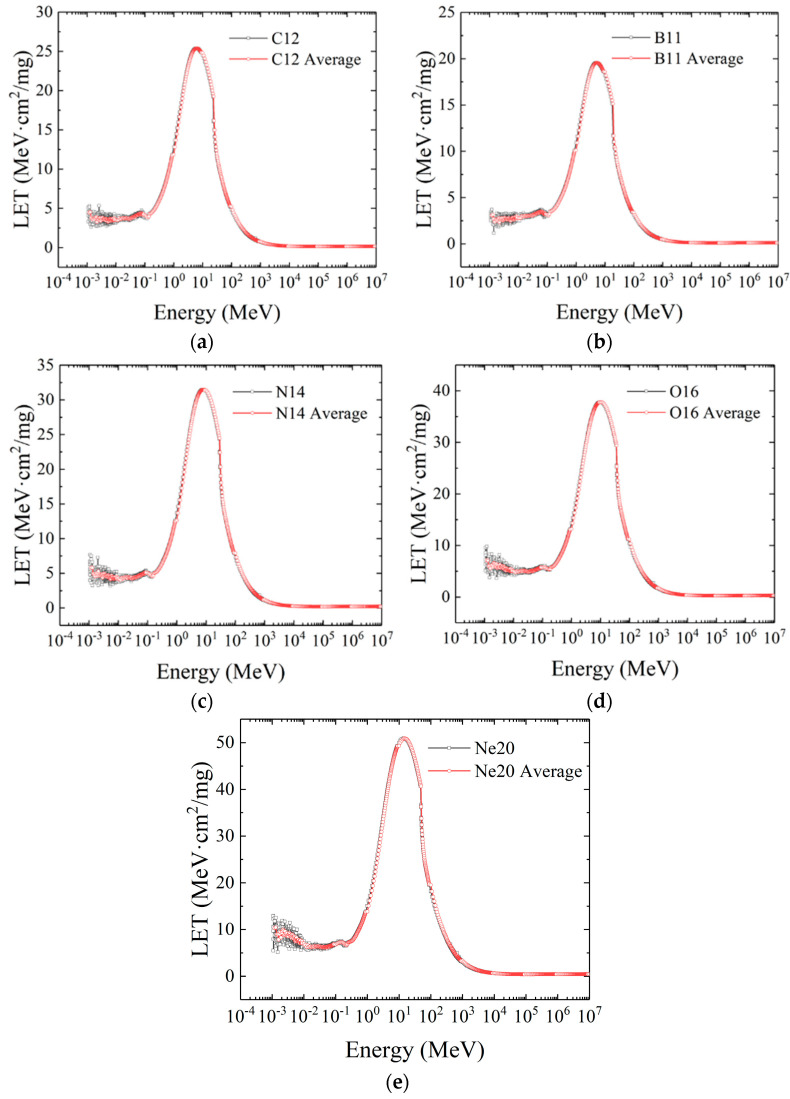
The LET distribution curves of B^11^ (**a**), C^12^ (**b**), N^14^ (**c**), O^16^ (**d**), and Ne^20^ (**e**) heavy ions.

The simulation extracts the LET of heavy ions with different initial energies, and 10 kinds of heavy ions are selected. Their atomic number Z and relative atomic mass A are shown in Table 2:

The first group of displayed curves includes the LET distribution curves of B^11^ (a), C^12^ (b), N^14^ (c), O^16^ (d), and Ne^20^ (e) heavy ions, as shown in Figure 5. When the atomic number is ≤20, the LET distribution curves of the four representative ions are relatively close. In the low-energy range of 10^−3^ MeV to 10^−1^ MeV, the LET values of all four ions fluctuate within a small range, and as the atomic number increases, the fluctuation becomes increasingly intense. In the energy range of 10^−1^ MeV to 10^1^ MeV, the LET values of the four ions increase rapidly in an approximately exponential distribution until reaching a peak. Additionally, the peak LET value increases continuously with the rise in atomic number, and the initial energy of the ions corresponding to the peak also increases incrementally.

**Figure 6:**
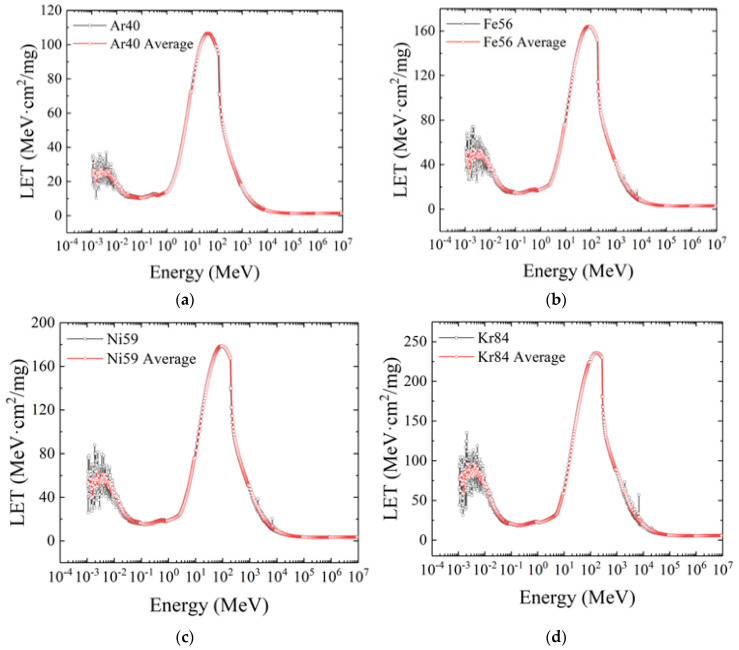
The LET distribution curves of (**a**) Ar40, (**b**) Fe56, (**c**) Ni59, and (**d**) Kr84 heavy ions.

The second group of displayed curves includes the LET distribution curves of Ar40, Fe56, Ni59, and Kr84 heavy ions, as shown in Figure 6. The LET distribution of the four kinds of heavy ions with medium atomic numbers is relatively consistent, and there are two peaks in the full spectrum range, which exist near the energy of 10^−3^ MeV to 10^−2^ MeV and 10^2^ MeV, respectively, and with the increase in atomic number, the two peaks tend to be close, which indicates that the LET increase rate in the low energy range is greater than that in the high energy range. This is because with the increase in atomic number, in addition to the energy deposition caused by the scattering of heavy ions and materials, secondary particles and secondary ionization are also produced. Similarly to the ions with a low atomic weight, the LET value of heavy ions with a medium atomic number gradually increases with the increase in atomic number, and the initial energy corresponding to the peak LET value also increases.

**Figure 7:**
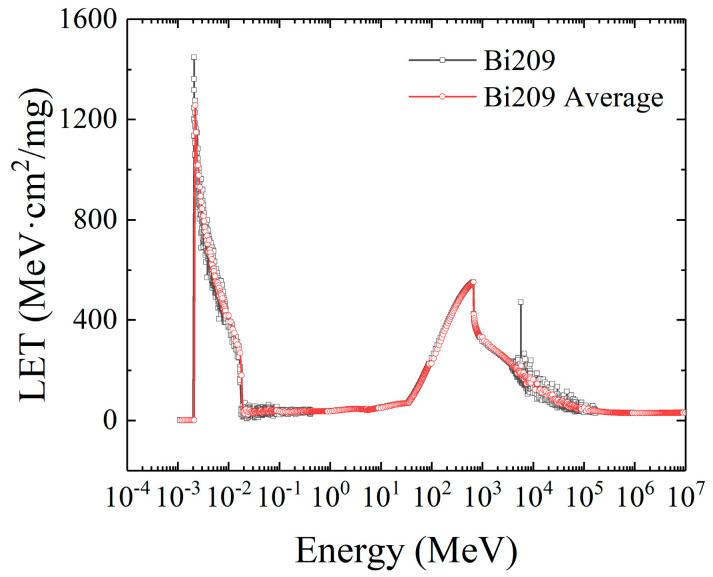
The LET distribution curve of Bi^209^.

The third curve is the LET distribution curve of Bi209, as shown in Figure 7.

The atomic number of Bi209 is 83, which belongs to the group of ions with a large atomic number in the range of 4~92 for heavy ions. It can be observed from its LET distribution curve that the LET distribution curve of Bi209 for heavy ions still shows the feature of double peaks, and the LET peak value in the low-energy-level range has exceeded that in the high-energy-level range. This is mainly due to the strong nuclear reaction between the heavy ions and the atoms of the target material, resulting in the generation of a variety of secondary particles, including alpha particles, gamma particles, electrons, and other heavy ions. These secondary particles collide with the atoms of other target materials, resulting in a sharp increase in the electron–hole pair, which is manifested as a sharp increase in the LET value of the low energy region.

**Figure 8:**
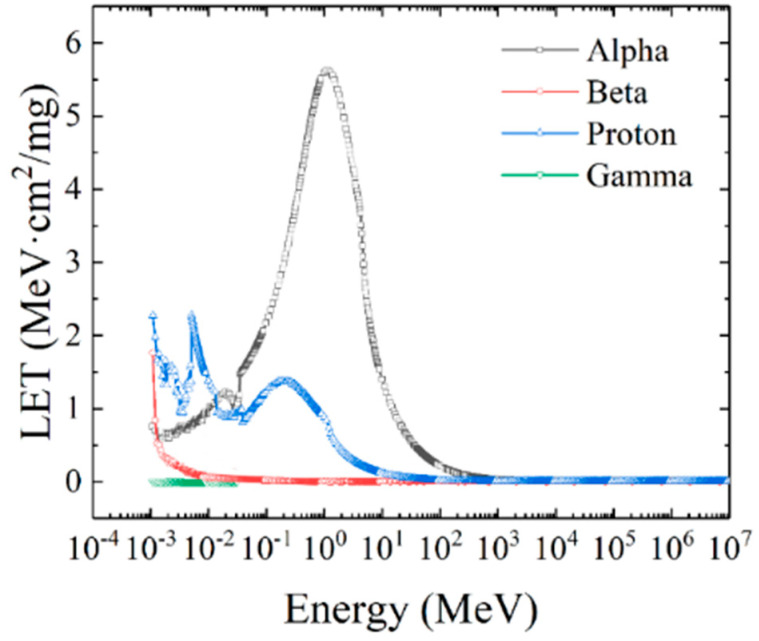
The average LET distribution of light particles.

**Figure 9:**
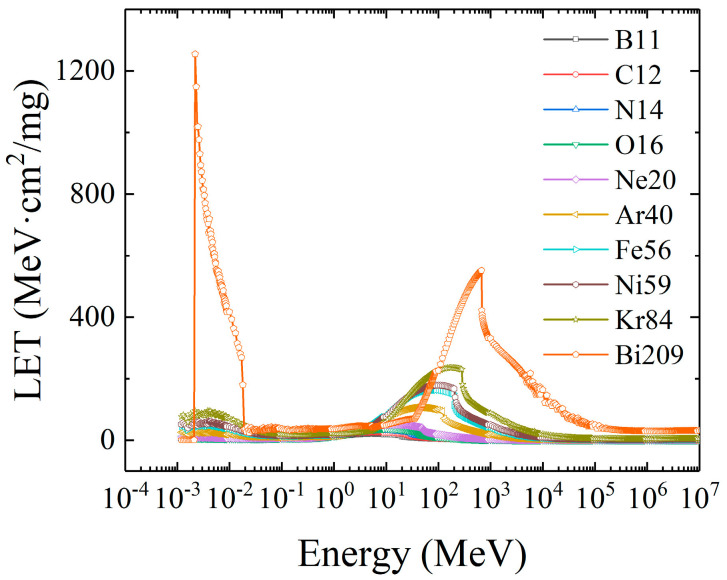
The LET value distribution curve of all heavy ions.

The distribution curve of LET linear mean value of all light particles is integrated, and the LET distribution curve of light particles in the full spectrum is shown in Figure 8:

It can be clearly observed from the figure that in the range of 10^−3^ MeV to 10^−2^ MeV, the average LET values of proton, alpha, beta and gamma particles decline successively. From 10^−2^ MeV, the LET values of alpha particles are significantly different from those of other particles. The average LET values of alpha, proton, and beta particles decreased sequentially, and after 10^3^ MeV, the LET values of all four ions decreased to stable values. In the whole energy range, the linear average LET value distribution curve of all heavy ions is shown in Figure 9. All heavy ions have peaks, respectively, in the low-energy-level region and the high-energy-level region, and the overall distribution is similar to that of double peaks. With the increase in atomic number, the double peaks become more obvious and gradually converge, and the peak number keeps increasing. The LET distribution of heavy ions in each energy range follows the law of increasing with the increase in atomic number, the LET value of the low energy level is similar to an exponential increase, and the LET value of the high-energy-level region is similar to the linear relationship with energy.

**Figure 10:**
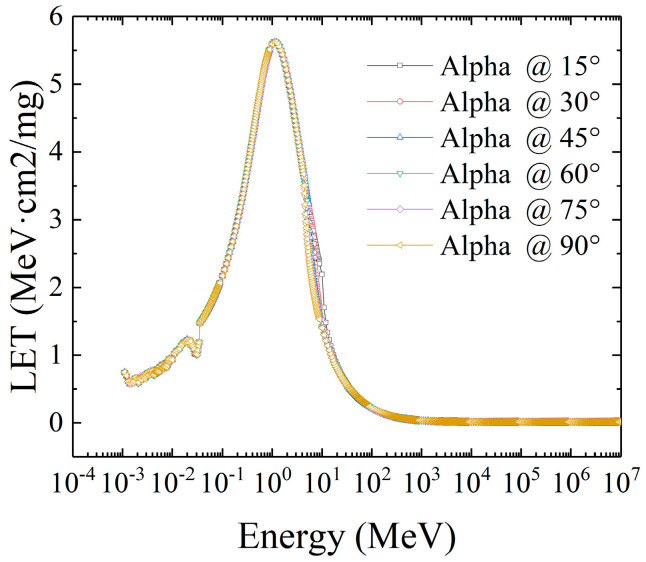
The distribution curve of average LET values for alpha incident at different angles.

The average LET values of alpha particles at different incident angles are shown in Figure 10. Within the full energy spectrum range, except that the LET curve near 10^1^ MeV is slightly different, it can be considered that the incident angle has no significant impact on the LET distribution of alpha particles.

The reason for this is that due to their large mass, alpha particles hardly deflect after collisions, and their tracks always remain “quasi-straight”; even when the incident angle is oblique, the number of collisions per unit length and the amount of energy transfer along the path are consistent with those under normal incidence.

## 4. Conclusions

In this paper, a SiC material model was established based on Geant4 simulation software, and the results of the single-incident LET distributions of different light particles and heavy ions with different atomic weights at different incident energies were simulated. The results showed that the LET value of protons oscillated significantly at low energy incidence, and gradually decreased exponentially after 10^−1^ MeV. The alpha particle has a LET peak near 1 MeV, the beta particle drops exponentially, and the gamma particles have little energy deposition, and almost pass directly through the material. The 10 kinds of heavy ions used in the simulation show peak values in the low- and high-energy-level regions, and the overall distribution is similar to that of double peaks. With the increase in the atomic number, the double peaks become more obvious and gradually converge, and the peak value keeps increasing. The LET distribution of heavy ions in each energy range follows the law of increasing with the increase in atomic number, the LET value of the low energy level is similar to an exponential increase, and the LET value of the high-energy-level region is similar to a linear relationship with energy. In the future, we will further study the interaction results of particles with different angles, different target materials, and different doping concentrations of the same target materials.

## Figures and Tables

**Table 1:** Definitions of different target materials.

Material	Length × Width (mm^2^)	Thickness	Density (g/cm^3^)
SiC	3 × 3	12 μm	3.2
SiO_2_	3 × 3	50 nm	2.2
Ti	1 × 1	50 nm	4.506
Al	1 × 1	50 nm	2.7

**Table 2:** Atomic numbers and relative atomic masses of heavy ions.

Heavy Ion	Z	A	Heavy Ion	Z	A
B	5	11	Ar	18	40
C	6	12	Fe	26	56
N	7	14	Ni	28	59
O	8	16	Kr	36	84
Ne	10	20	Bi	83	209

## Data Availability

The original contributions presented in this study are included in the article. Further inquiries can be directed to the corresponding author.
